# The Treatment of Patients Found Unconscious

**Published:** 1907-06-22

**Authors:** 


### RESIDENT MEDICAL OFFICERS' DEPARTMENT.

[Contributions to this Column are invited, and, if accepted, will be paid for.]

### THE TREATMENT OF PATIENTS FOUND UNCONSCIOUS.

THERE exists in the police force, and rightly so, a rule to take to a hospital all people found lying unconscious on doorsteps, pavements, or in public places. Frequently a constable witnesses an obviously drunk and disorderly person reel and fall, and removes him directly to the police station, whither, if necessary, the divisional surgeon can be summoned; but the "found drunk" cases come under the regulation and are brought to hospital. In hardly any other circumstance is the house physician or house surgeon likely to meet so many pitfalls; from the moment of the patient's arrival to that of his departure or admission into a ward his medical attendant must maintain an entirely unbiassed attitude.

The temptation to assume the probable is in the majority of instances so overpowering that the long gamut of the possible is too often neglected. The signs of simple drunkenness, from the early stages of slightly flushed face and confused loquacity up to those of delirious excess, in manner and in language, together with an odour of some alcoholic beverage, are too familiar to call for detailed comment. It is when the stuporous condition has supervened that mistakes in diagnosis are difficult to avoid and serious in their consequences. In the large London hospitals an unconscious man without obvious injury comes under the care of the house physician, but if a cut head (or any injury, however slight) is noticed he falls to the lot of the house surgeon. This emphasises the importance of examining the entire body of any patient under the influence of drink. A deeply drunken man is anæsthetic, and a series of fractured ribs or even a fractured skull may pass unnoticed, because there is no evidence of pain. Every joint, bone, and viscus should be examined by routine to eliminate the presence of injury or disease.

It is a safe rule to believe a man sober until he is proved to be drunk, and the varying intensity of alcoholic effect, from the mere stimulation of the animal spirits to the sodden bestiality of dead drunkenness, must be kept in mind. Further, the medical officer must not forget that the condition may be one simulating drunkenness, or complicated by drunkenness, instead of mere drunkenness *per se*. Brandy, being the most easily obtained stimulant, is given so frequently in cases of faintness by friends and even forced between the teeth of unconscious people before arrival at hospital, that it is always well to inquire if this form of first-aid has been adopted. The masking effect of alcohol is apt to escape recollection, and the sin of commission of the patient may tempt the doctor to the sin of omission. Definite physical injuries apart, it is next necessary to exclude the more common causes of unconsciousness, partial or complete. Ale-drinkers form so large a percentage of the population, and their breath is so often heavy with the fumes of beer, that it is not necessary to presuppose more than the usual meal just previous. The smell of opium may be covered, but, as in cases of cerebral hæmorrhage, the condition of the pupil will be of help. Muscular movements of the limbs and head and trunk must be noted, the condition of the reflexes determined, and the ingravescent comatose condition of intracranial hæmorrhage remembered. The unconscious states in diabetic coma, in the post-epileptic condition, and in albuminuric coma must be kept in mind. In all cases of doubt the urine must be examined and the lungs overhauled; in any case a distended bladder should be relieved. It is beyond the scope of this article to enter into a differential diagnosis of the comatose conditions produced by poisons; it will be sufficient to mention opium, prussic acid and the cyanides, the chloral group, and ptomaines.

When everything but drunkenness has been deemed absent, and the house officer has decided that alcohol alone is the cause of the condition, the question of treatment resolves itself along easy lines. When the diagnosis is uncertain or complicated, expectant treatment must be adopted. Assuming that the patient is absolutely and helplessly drunk, and that he is known to the police as an habitual drunkard, he may be handed over to their care. But even in this case caution is advisable, and it is in almost every instance the safest plan to keep the patient for some time and watch his progress at intervals. In the meantime, to aid recovery, the stomach should be emptied. The usual emetics act but feebly on a full stomach whose walls are distended and anæsthetic; moreover, drugs by the mouth may be difficult to give; on the other hand, the process of washing out the stomach is tedious and unpleasant. The quickest and most certain method is to inject $\frac{1}{15}$ to $\frac{1}{10}$ gr. apomorphine hydrochloride under the skin of the arm. This acts very thoroughly. The return to consciousness may be greatly facilitated by a judicious use of the battery.

It is never wise to humour the eagerness of the police for a swift diagnosis. The resident may do one of three things: he may say that the patient is "under the influence of alcohol" if he is only tipsy, and send him home in charge of the police; or he may send one constable for the friends, keeping the other until they come, and then, dismissing the police, retain the friends until soberness returns. Thirdly, the offensively and uproarously "drunk," who is intractable and cannot be helped, must perforce be sent to the cells, to be fined or imprisoned on the morrow. To sum up, the best plan, alike for the patient, the hospital, and the house officer, is to examine exhaustively, to diagnose warily, and to treat expectantly, always erring on the safe side.

